# Dissecting protein loops with a statistical scalpel suggests a functional implication of some structural motifs

**DOI:** 10.1186/1471-2105-12-247

**Published:** 2011-06-20

**Authors:** Leslie Regad, Juliette Martin, Anne-Claude Camproux

**Affiliations:** 1 INSERM, U973, Paris F-75013, France; 2 Université Paris 7 - Paris Diderot,UMR-S973, MTi, F-75013 Paris, France; 3 Université Lyon 1, Univ Lyon, France; CNRS, UMR 5086; Bases Moléculaires et Structurales des Systèmes Infectieux, IBCP 7 passage du vercors, F-69367, France

## Abstract

**Background:**

One of the strategies for protein function annotation is to search particular structural motifs that are known to be shared by proteins with a given function.

**Results:**

Here, we present a systematic extraction of structural motifs of seven residues from protein loops and we explore their correspondence with functional sites. Our approach is based on the structural alphabet HMM-SA (Hidden Markov Model - Structural Alphabet), which allows simplification of protein structures into uni-dimensional sequences, and advanced pattern statistics adapted to short sequences. Structural motifs of interest are selected by looking for structural motifs significantly over-represented in SCOP superfamilies in protein loops. We discovered two types of structural motifs significantly over-represented in SCOP superfamilies: (i) ubiquitous motifs, shared by several superfamilies and (ii) superfamily-specific motifs, over-represented in few superfamilies. A comparison of ubiquitous words with known small structural motifs shows that they contain well-described motifs as turn, niche or nest motifs. A comparison between superfamily-specific motifs and biological annotations of Swiss-Prot reveals that some of them actually correspond to functional sites involved in the binding sites of small ligands, such as ATP/GTP, NAD(P) and SAH/SAM.

**Conclusions:**

Our findings show that statistical over-representation in SCOP superfamilies is linked to functional features. The detection of over-represented motifs within structures simplified by HMM-SA is therefore a promising approach for prediction of functional sites and annotation of uncharacterized proteins.

## Background

Protein structures can usually be broken down into their component secondary structures: *α*-helices, *β*-strands and loops. *α*-helices and *β*-strands are regular secondary structures recurrent in many proteins. Protein loops correspond to all residues not assigned to regular secondary structures. Unlike *α*-helices and *β*-strands, protein loops were initially seen as random coils because their sequences and structures are highly variable. But the ever-increasing availability of protein structures in the Protein Data Bank (PDB) allowed extensive analyzes of protein loops, which suggested a more complex view. For example, Panchenko *et al*. [1] analyzed the evolution of protein loops and identified a linear correlation between sequence similarity and mean levels of structural similarity between loops in protein families. They suggested that loops evolve through a process of insertion/deletion and concluded that even longer loop regions cannot be defined as irregular conformations or random coils. Several classifications of short and medium loops have been developed [2-7], according to the type and structure of flanking secondary structures, and the length and geometry of loops. These classifications have revealed the existence of recurrent amino-acid dependent loop conformations.

Loop regions play a role in protein function [8]. They may be involved in the active sites of enzymes [9] or in binding sites [10-13]. The classification of protein loops has then been used to investigate the link between protein loops and function. From the loop classification system ArchDB [3], Espadaler *et al*. [14], developed an approach to identify loop clusters associated with the protein functional sites provided by the PROSITE database [15] or Gene Ontology (GO) [16]. They showed that loops contain structural motifs involved in the functional sites of proteins. Using a similar approach, Tendulkar *et al*. [17] and Manikandan *et al*. [18] extracted octapeptide clusters involved in protein function. They first classified octapeptides using geometric invariants [17] or dihedral angles [18]. They then identified octapeptide clusters associated with protein functions provided by SCOP superfamilies [19] or GO terms. Tendulkar *et al*. found that functional clusters consisted mostly of octapeptides extracted from loop regions [17]. In a similar vein, Polacco *et al*. [20] developed the GASPS approach (Genetic Algorithm Search for Pattern in Structure) to extract the structural motifs most useful for identifying SCOP superfamilies. Ausiello *et al*. [21] developed an approach called FunClust to identify conserved residues of three-dimensional (3D) structural motifs through local structural comparisons between non homologous proteins. The common point between all these studies is that no prior information about the location of the functional sites is required, making it possible to discover new functional sites.

Contrary to the methods cited above, other approaches start from known functional sites and look for structural motifs associated with them [22-26]. In all these approaches, structural motifs are learned through structural alignment [27], conservation of environment [26,28], or calculation of geometrical parameters [22-24]. The goal, here, is different than the one pursued by classification studies: since the focus is set on known functional sites, these approaches are dedicated to the prediction of these known functional sites, not to the discovery of new sites with functional implication.

There is a third family of studies that we need to introduce before presenting our work: the identification of functional sequential motifs in DNA sequences using pattern statistics. The strategy consists in searching for nucleotide motifs with unusually high or low frequencies, i.e. over- or under-represented, with respect to a reference model (generally a homogeneous Markov model) [29,30]. The underlying idea is that the unusual frequency of a sequence motif in a genome reflects a selective pressure on this motif, suggesting a functional role. Such studies have led to the successful identification of functional motifs, such as restriction sites [31], cross-over hotspot instigator sites [32] and polyadenylation signals [33].

In this paper, we propose an approach inspired by this last category of studies to identify structural motifs in loops involved in protein function. Our approach is based on two components. The first one is the structural alphabet HMM-SA described in [34-37]. It is a collection of 27 structural prototypes of four residues, called structural letters, connected by transition rules. HMM-SA allows simplifying protein 3D structures into one-dimensional (1D) sequences of structural letters. After this simplification step, the search for 3D structural motifs is reduced to the search for structural words in the 1D structural-letter sequences. We can then apply the second component of our approach: the SPatt software that allows computing exact statistics in short sequences [38], which we use to detect over-represented structural words. We specifically focus on structural motifs of seven residues in loops, following the protocol developed in [39]. In this previous publication, we have shown that this protocol allowed grouping together seven-residue fragments with very similar structures, extracted from both short and long loops [39]. An advantage of this method is that it does not require pairwise comparison of all seven-residue fragments. In this study, we further investigate the functional implication of over-represented structural motifs. We consider the SCOP classification at the superfamily level, which groups protein with similar functions. For every structural word, we compute the over-representation separately in each SCOP superfamily. Based on the statistical over-representation in SCOP superfamilies, we make the distinction between two types of over-represented structural words within loops: structural words over-represented in multiple superfamilies, called ubiquitous words, and structural words over-represented in one or few superfamilies, called superfamily-specific words. To assess the role of these words, we (i) investigate the correspondence between a subset of ubiquitous words and known recurrent motifs, such as turns and niches and (ii) check the link between a subset of superfamily-specific words and functional sites of proteins, provided by Swiss-Prot functional annotations. This validation step confirms that superfamily-specific words are involved in some functional sites of proteins, such as the binding sites of small ligands. Our method thus allowed the identification of structural motifs important for protein function. Some were previously known as involved in protein functions, others are new structural motifs with a putative functional role. Our results indicate that our statistical approach is a promising approach for the detection of new structural motifs of interest in protein structures.

## Methods

### Protein data sets

#### Initial data set

A list of 8 119 protein structures was extracted from the PDB of May 2008 with PISCES software [40], using the following criteria: data obtained by X-ray diffraction, with a resolution better than 2.5 Å, longer than 30 residues, less than 50% sequence identity between any pair. We restricted this list to the 5 429 structures classified in SCOP [19]. As it is assumed that proteins grouped in the same SCOP superfamily have similar structure and function, this level was chosen for our analysis. For statistical analysis, we further restricted the list to proteins classified into superfamilies with at least two members in the data set, corresponding to 4 911 proteins from 1 493 superfamilies. On average, a superfamily contains 7.90 proteins (±13.78).

#### Annotation data set

To validate the functional role of over-represented structural words, we analyzed their correspondence with functional annotations extracted from the Swiss-Prot database. Swiss-Prot is a curated sequence database providing a high level of annotation (description of protein function, domain structure, post-translational modifications, variants, etc.), a minimal level of redundancy and a high level of integration with other databases [41]. To extract functional annotations from our initial data set, we used the PDB/UniProt Mapping database [42], which consists of several files mapping the PDB and UniProt codes, and PDB and UniProt sequence numbering. Only 1 487 of the 4 911 protein structures of our initial data set are present in the PDB/UniProt Mapping database. From this set of 1 487 proteins, called annotation data set, we extracted the Swiss-Prot annotations. We focused on the feature table listing post-translational modifications, binding sites, enzyme active sites, local secondary structure or other features. We extracted only the following annotations: "Repeat" (Positions of repeated sequence motifs or repeated domains), calcium, DNA, nucleotide-binding sites, metal-binding sites (cobalt, copper, iron, magnesium, manganese, molybdenum, nickel, sodium), zinc finger, active sites, and binding sites for any chemical group (co-enzyme, prosthetic group, etc).

#### Validation data set

This data set was used to double-check the correspondence between structural motifs and Swiss-Prot annotations. From PDB/UniProt Mapping database, we extracted a set of 2 640 proteins classified in SCOP. From this protein set, we retained the 2 636 proteins obtained by X-ray diffraction, with a resolution better than 3 Å, longer than 40 residues and presenting less than 95% sequence identity between any pair.

### Extraction of over-represented structural motifs from protein loops

**Figure 1:**
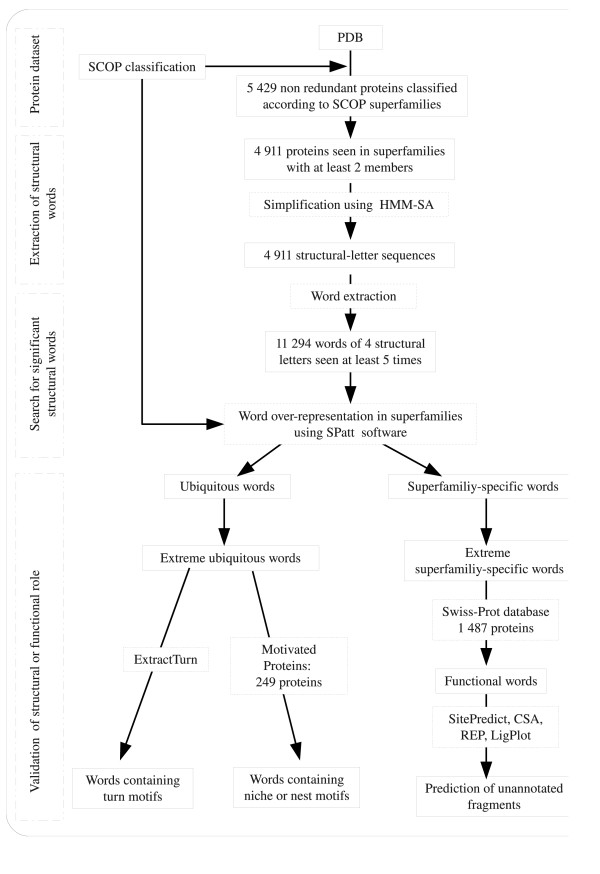
**Protocol used in this study**. Non redundant protein structures were simplified using the structural alphabet HMM-SA and structural motifs extracted using the protocol presented in Figure 2. Over-represented structural motifs in SCOP superfamilies in protein loops were detected using the SPatt software. Based on SPatt statistics, two types of words were distinguished: ubiquitous words, over-represented in several superfamilies, and superfamily-specific words, over-represented in few superfamilies. Some ubiquitous words were compared with known structural motifs: *β*-turns identified by the ExtractTurn software and structural motifs presented in the Motivated Proteins database. Some superfamily-specific words were compared with functional sites, using Swiss-Prot annotations and external softwares.

Our approach, summarized on Figure 1 is based on two components: (i) the structural alphabet HMM-SA that allows the simplification of protein structures into structural-letter sequences, (ii) the SPatt software that allows the computation of exact pattern statistics in simplified structural-letter sequences. We describe below these two components.

#### Simplification of protein structures by HMM-SA and extraction of structural motifs

HMM-SA is a structural alphabet of 27 structural prototypes of four residues, called structural letters, established with hidden Markov models. The main steps of HMM-SA construction are the following (see [34,36] for details):

1. the backbone of protein structures of a large data set are split in overlapping fragments of four residues,

2. each four-residue fragment is described by the three distances between the non-successive *α*-carbons and the projection of the fourth α-carbon on the plan formed by the first three ones,

3. four-residue fragments are classified according to their geometry and their succession in protein structures, using a hidden Markov model where the inputs are the vectors of distance descriptors of each fragment.

4. the optimal structural alphabet model is selected using the parsimony principle to choose the model that better fits the data with the smallest possible complexity. In this goal, structural alphabets of different lengths are compared using the Bayesian Information Criterion, which balances the log-likelihood of the model and a penalty term related to the number of parameters of the model and the sample size.

**Figure 2:**
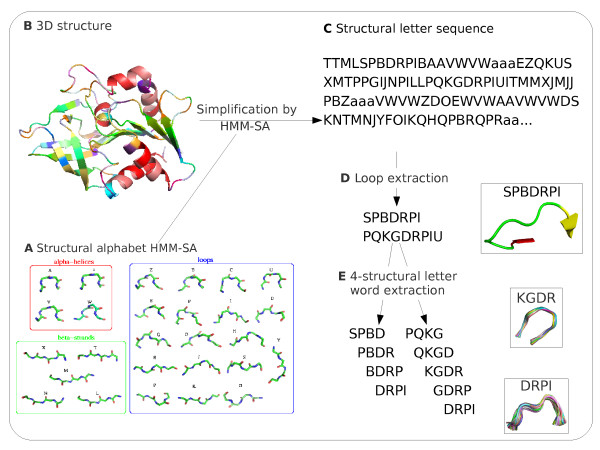
**Protocol used for extraction of structural motifs**. A: the 27 structural letters of HMM-SA. B: input 3D structure. C: sequence of structural letters resulting from the simplification. D: extraction of loops based on regular expressions of structural letters; the geometry of a loop encoded by SPBDRPI is shown on the right side. E: systematic splitting of loops into overlapping words of four consecutive structural letters. The geometry of two structural words, KGDR and DRPI, are shown with superimposition of their fragments. Fragments are superimposed with ProFit software http://www.bioinf.org.uk/software/profit and represented with Pymol http://www.pymol.org.

The optimal HMM-SA resulted in 27 classes of four-residue fragments and the transition matrix between these classes. For each class, labelled by letters (a, A-Z) and named structural letters, a representative four-residue fragment, presented in Figure 2A, is computed. It has been shown that four structural letters (A, a, W, V) are specific to *α*-helices, five (L, M, N, T, X) are specific to *β*-strands and the remaining 18 describe loops [36].

HMM-SA can be used to simplify a protein structure of *n *residues into a sequence of (*n *- 3) structural letters. This simplification takes into account the structural similarity of four-residue fragments with the 27 structural letters. It is achieved by a dynamic programming algorithm based on Markovian process to obtain maximum *a posteriori *encoding using the Viterbi algorithm. The input is the sequence of distance descriptors of the four-residue fragments of the input structure. The output is a sequence of structural letters, where each structural letter describes the geometry of a four-residue fragment.

We used HMM-SA to extract structural motifs from protein loops using the protocol established in a previous study [39] and summarized in Figure 2. We first simplified all the 4 911 structures of our initial data set in sequences of structural letters. Since we focused our analysis on protein loops, regular secondary structures were removed, based on the fact that some structural letters are specific to regular secondary structures [36,37]. From the initial data set, we obtain 90 811 protein loops encoded into structural-letter sequences. In these 90 811 protein loops, we chose to study the structural motifs formed by four consecutive structural letters (i.e., seven residues). The choice of the length of four structural letters is motivated by our previous work [39], where we showed that it allows a compromise between considering long fragments on the one hand, and avoiding data sparsity on the other hand. The 90 811 protein loops are split into 238 158 seven-residue fragments, described by 25 304 different words of four structural letters. As we have previously shown that structural words with low frequencies are linked to structural flexibility and regions with uncertain coordinates [39], we did not consider structural words seen less than five times in our initial data set. This results in a set of 11 294 different structural words, grouping 224 148 seven-residue fragments. Each word is seen on average 20 times (±32), meaning that it groups on average 20 seven-residue fragments.

#### Computation of pattern statistics using SPatt

We used the SPatt software [38,43], available from http://stat.genopole.cnrs.fr/spatt/index.html to identify structural motifs over-represented in SCOP superfamilies.

**Figure 3:**
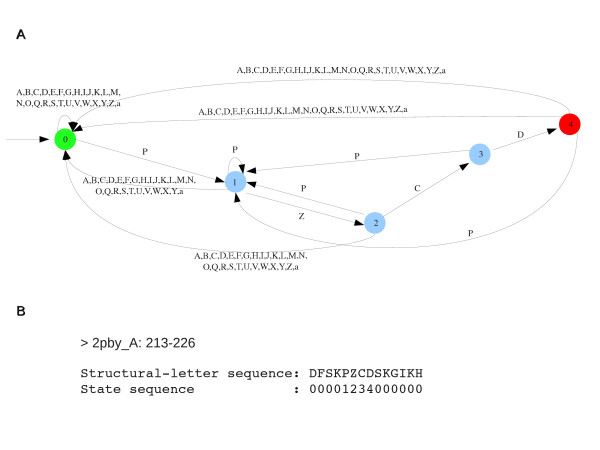
**Example of Markov chain embedding for the **PZCD**pattern**. A: Deterministic Finite Automaton (DFA) associated to PZCD. Initial state is highlighted in green, transiting states in blue and final state in red. One proceeds in this DFA according to the labels associated to the rows between states. Each occurrence of PZCD will reach the final state. B: state sequences obtained after passing of a structural-letter sequence to the DFA.

Here, we computed the over-representation of four-structural-letter motifs in sets of protein loops grouped by SCOP superfamilies. The considered sequences are typically short. The SPatt approach allows the calculation of exact statistics in sets of short sequences [44,45]. The over-representation of a word *w *in a set of sequences is assessed by comparing its observed occurrence (*N_obs_*) with the theoretical occurrence (*N_theo_*) expected under a background model. The over-representation score *Lp *of *w *is given by(1)

where the *p *- *value *is defined by:(2)

where *P *denotes the probability of the events. For instance, a *Lp *score of 3 means that a word is over-represented with a *p *- *value *of 10^-3^. SPatt allows the exact computation of the distribution of the word occurrence *N_theo _*and thus the corresponding *p *- *value*. The approach implemented in SPatt is based on the notion of automata. We briefly present it below, see [44,45] for details. Let us consider, for example, the word PZCD. The first step in SPatt consists in building an optimal Markov chain embedding through a Deterministic Finite Automata (DFA) shown in Figure 3A. The second step in SPatt consists in passing the structural-letter sequences in the DFA, resulting in the corresponding state sequence as illustrated in Figure 3B. By definition these state sequences are a heterogeneous first order Markov chain embedding over the alphabet , with a starting distribution *m_d _*(*d *∈ [1, *r*]) and a transition matrix *T*. The computation of *m_d _*and *T *are explained in [44]. Then, these corresponding Markov chain embedding parameters allow the computation of the generating function of *N_w _*in each structural-letter sequence. From the generating functions, , of *N*_*the*o_, all terms of equation 1 are deduced, see [44]:(3)(4)

A simple example of the computation of *p - value *of word using DFA is presented in details [44]. Note that, contrary to approaches based on the hypergeometric distribution approximation, the exact approach does not require any correction to take into account the size of the data set in which the patterns are searched. This is explicitly taken into account during the exact *p - value *computation.

In this work, we computed the over-representation scores for four structural-letter words, in the loop regions of proteins classified into SCOP superfamilies. In each of the 1 493 superfamilies, we computed the *Lp *scores of those words, among the 11 294 that meet the condition of being observed at least five times in the superfamily. In order to take into account multiple testing, we used the Bonferroni correction to set the significance threshold, resulting in a final threshold equal to 5.97.

We further considered two criteria:

• *Lp_max_*: the maximal *Lp *score of a word among all superfamilies,

• nb_sf*_: the number of superfamilies in which a word is significantly over-represented.

These two criteria enabled us to differentiate two types of over-represented structural words, as defined in Table 1: words over-represented in a large number of SCOP superfamily, with *Lp_max _*> 5:97 and nb_sf* _>= 5, which we refer to as *ubiquitous words *and highly over-represented in one superfamily, with *Lp_max _*> 5.97 and nb_sf* _< 5, which we refer to as *superfamily-specific words*.

**Table 1:** Definition of word types

Name	Definition
Structural word	Sequence of four successive structural letters

Over-represented word	Structural word with *Lp_max_* ≥ 5.97

Ubiquitous word	Structural word with *Lp_max _*≥= 5.97 and *nb*_sf* _≥= 5

Extreme ubiquitous word	Structural word with *Lp_max _*≥= 10 and *nb*_sf* _≥= 5

Superfamily-specific word	Structural word with *Lp_max _*≥ 5.97 and *nb*_sf* _< 5

Moderately superfamily-specific	Structural word with *Lp_max _*≥= 10 and *nb*_sf* _< 5

Extreme superfamily-specific word	Structural word with *Lp_max _*≥= 50 and *nb*_sf* _< 5

Functional word	Extreme superfamily-specific word with a precision≥ 40% for a Swiss-Prot annotation

*: extreme structural words are subject to further examination to validate their structural or functional role.

For comparison, we also calculated these criteria over randomized data sets obtained by randomly reassigning loops to SCOP superfamilies.

### Extent of coverage of structural words

**Figure 4:**
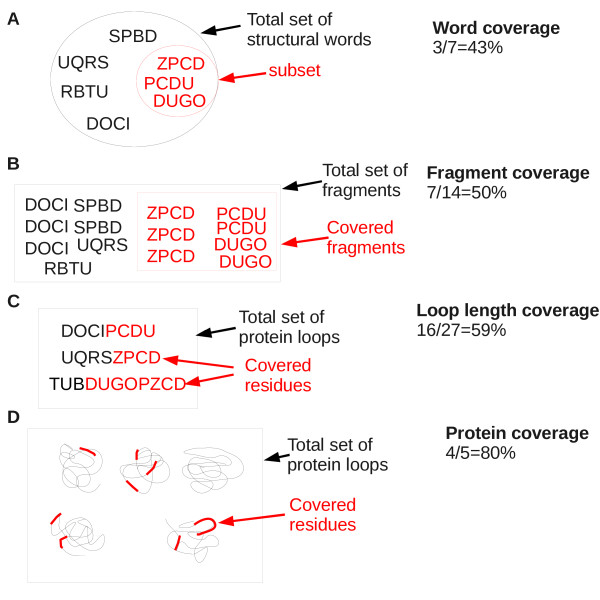
**Definitions and illustration of coverage rates**. We considered a set of seven words of four structural letters (SPBD, UQRS, RBTU, DOCI, ZPCD, PCDU, DUGO), grouping 14 seven-residue fragments. Let us consider that these words and their occurrence are examples and not the real occurrences in the data set. From this set of words, we focused on three words, named restricted set and presented in red in A, grouping seven seven-residue fragments. Various coverage rates were calculated for these words. A: word coverage, the fraction of structural words included in the restricted set. B: fragment coverage, the fraction of fragments encoded by words from the restricted set. C: loop-length coverage, the fraction of residues in loops covered by words from the restricted set. D: protein coverage, the fraction of proteins containing at least one of the words from the restricted set.

Let us consider a data set of protein structures encoded in structural-letter sequences and a subset of structural words. The coverage of the data set by the subset of structural words can be calculated at various aspects, illustrated in Figure 4:

• word coverage: the fraction of structural words included in the word subset,

• fragment coverage: the fraction of fragments encoded by words from the subset,

• loop length coverage: the fraction of residues in loops covered by words from the subset,

• protein coverage: the fraction of proteins containing at least one of the words from the word subset.

### Validation of structural or functional role of structural words

Our protocol enabled us to extract over-represented structural motifs in from loops. Then, we tried to assess the implication of these words in a structural or a functional point of view. Specifically, we investigated (i) the link between ubiquitous words and known structural motifs and (ii) the link between superfamily-specific words and known functional sites. This step of validation was performed on the annotation and validation data sets, only for a subset of the most significantly over-represented structural words, called extreme words, as defined in Table 1.

#### Validation of the structural role of extreme ubiquitous words

Ubiquitous words were compared with well-characterized 3D motifs: *β*-turns, niche and nest motifs. *β*-turns are detected in protein structures with ExtractTurn software [46]. Turns are defined as tetrapeptides with an  distance lower than 7 Å, with the two central residues *i *+ 1 and *i *+ 2 in a non helical state [47]. Nest and niche motifs are identified using the Motivated Proteins database [48]. Nest motifs are fragments of three consecutive residues, in which the main-chain NH of residue *i *and the main-chain NH of residue *i *+ 2 have the potential to interact weakly with an anionic group [49]. Niche motifs are formed by three or four consecutive residues in which the main-chain CO of residue *i *and the main-chain CO of the last residue *i *+ 2 or *i *+ 3 have the potential to interact weakly with a cationic group [50]. The Motivated Protein database stores the nest and niche motifs detected in a data set of 400 representative proteins. Only 249 of these 400 proteins are also included in our initial data set. The comparison of structural words with nest and niche motifs is thus restricted to these 249 proteins. The Motivated Protein database was also used to detect ends of *β*-turns. For a pair formed by a structural word and a known structural motif, we computed a precision measure given by the proportion of fragments encoded by the structural word that contain the known structural motif.

#### Validation of the functional role of extreme superfamily-specific words

The functional implication of superfamily-specific structural words was explored using the biological annotations from the Swiss-Prot database extracted from the annotation data set. The comparison of structural words with Swiss-Prot annotations extracted from annotation data set is limited to the 1 487 proteins. In an effort to limit this gap, we built a second data set, named validation data set composed of 2 636 proteins and favoring the selection of annotated proteins.

In order to quantify the correspondence between structural word and biological annotations, we computed precision and sensitivity measures of the detection of annotations using words. We considered two levels of annotation: the first level, named annotation, corresponds to the "Feature key" and the second level, named second-level annotation, corresponds to the "Description" that provides a description of the annotation. For example, when the annotation is "binding", the second-level annotation indicates the ligand type.

The precision is defined as the proportion of fragments encoded by a structural word that are annotated by a given annotation considering the two levels of annotation. A structural word with high precision is said to be functional. In order to take into account the sparsity of Swiss-Prot annotations, we set a permissive threshold of 40% precision. The sensitivity (also called recall) is defined by the proportion of a given annotation that is covered by a structural word. To compute the sensitivity, we retained only annotations extracted from protein loops, annotations seen in regular secondary structures regions are discarded.

In complement to Swiss-Prot annotations, which are of high quality but far from complete, we used various external tools to identify putative functional motifs.

• The Catalytic Site Atlas (CSA) database [51] documents enzyme active sites and catalytic residues in enzymes of known 3D structure. It identifies the residues directly involved in the enzymatic reaction.

• The Ligplot software [52] allows the identification of interactions between proteins and ligands, by providing schematic diagrams of protein-ligand interactions from a given PDB file.

• The REP software [53] is used to predict repeat regions from protein sequences. This software uses an iterative homology-based repeat finding method.

• The SitePredict software [24] http://sitepredict.org/ is used to predict nucleotide and calcium-binding sites. SitePredict is a machine learning method based on diverse residue properties, including the spatial clustering of residue types and conservation during evolution. Only residues with a score above 0.5 are considered to be involved in the binding site.

## Results

### Extraction of structural motifs over-represented in SCOP superfamilies

The goal of our study is to systematically identify structural motifs of interest, i.e. motifs with structural or functional implication, in protein loops. We made the hypothesis that structural motifs of interest are subject to selective pressure during evolution, which should result in structural words with unexpectedly high frequency in protein structures simplified into structural-letter sequences. In order to make the connection with protein function, we surveyed the over-representation of structural words in SCOP superfamilies, by computing over-representation scores for all structural words seen at least five times in a SCOP superfamily.

We counted a total of 1 705 structural words over-represented in at least one SCOP superfamily in the initial data set, corresponding to a coverage rate of 15% of the words and 30% of the fragments, as reported in Table 2. Based on the over-representation in SCOP superfamilies, we built two statistical criteria to classify the structural words: *Lp_max_*, which is the maximum over-representation score *Lp *observed among SCOP superfamilies, and nb_sf* _indicating the number of superfamilies in which a structural word is over-represented. For example, structural word GSUS has a *Lp_max _*value equal to 140 and a *nb*_sf* _value equal to 3, meaning that this word is over-represented in three SCOP superfamilies and very strongly in one of them with a *Lp *score equal to 140, i.e. a *p - value *equal to 10^-140^. Average values observed for *Lp_max _*and nb_sf* _are reported in Table 3. Globally, structural words display an average *Lp_max _*equal to 4.3 ± 5.6, with extreme values observed for the words PCDS (*Lp_max _*= 0.39) and UODO (*Lp_max_*= 210). The mean value of nb_sf* _is equal to 0.2 ± 0.7, ranging from 0 to 25, indicating that many of these words are not exceptional in any superfamily. We assessed the relevance of these numbers by comparing them with those obtained with randomized SCOP classifications. The number of over-represented words using random SCOP classifications is significantly smaller than that for SCOP: only 47 words are over-represented for the random SCOP classification, see Table 3. We can therefore conclude that over-represented words significantly depart from random regarding their repartition in SCOP superfamilies. 

**Figure 5:**
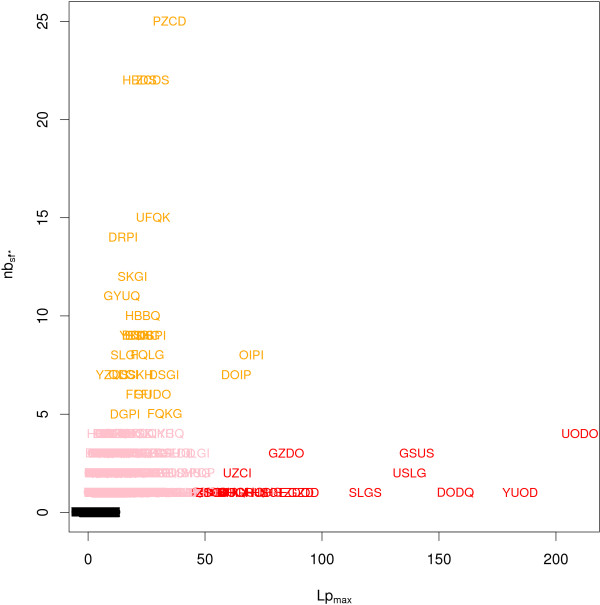
Plot of statistical criteria *Lp_max _*and nb_sf* _for the structural words seen at least five times in a SCOP superfamily. Black: words with *Lp_max _*≤= 5.97. Red: extreme superfamily-specific words (*Lp_max _*≥50 and nb_sf* _> 5). Orange: extreme ubiquitous words (*Lp_max _*≥10 and nb_sf* _≥ 5). Pink: over-represented words with *Lp_max _*> 5.97 not discussed in this study.

Figure 5 presents the values of Lp_max _versus nb_sf* _for all structural words seen at least five times in a SCOP superfamily. Interestingly, this representation reveals that some structural words are over-represented with very high scores in a small number of superfamilies, whereas others are over-represented with more moderate scores but in several superfamilies. Accordingly, we define two classes of words: ubiquitous and superfamily-specific words, as detailed in Table 1. Ubiquitous words are over-represented in several superfamilies, suggesting that they may be involved in protein structures. By contrast, superfamily-specific words are over-represented in few superfamilies, suggesting a possible association with functional sites. We then carried out an analysis of (i) the link between ubiquitous words and known recurrent structural motifs, and (ii) the link between superfamily-specific words and functional sites in proteins. This analysis was carried out only for a subset of the ubiquitous and superfamily-specific words, the *extreme *ubiquitous words and *extreme *superfamily-specific words as detailed in Table 1.

**Table 2:** Coverage rate (%) of different word subsets in the initial data set

Word subset	Number of words	Word coverage	Fragment coverage	Loop-length coverage	Protein coverage
Over-represented	1705	15	30	44	61
Extreme ubiquitous	24	0.2	3.4	5	63
Extreme superfamily-specific	23	0.2	0.7	1	17

Relaxed ubiquitous	40	0.4	4.5	6.5	72
Moderately superfamily-specific	114	1	3	5	77

**Table 3:** Statistics for the various word subsets

Data set	Word subset	Word number	Lp*_max_*	nb_sf*_
Initial data set	All words	11 294	4.3 (5.6)	0.2 (0.7)
	Over-represented words	1 705	11.3 (12.1)	1.3 (1.4)
	Extreme ubiquitous words	23	26 (14)	10.33 (5.5)
	Extreme superfamily-specific words	24	89 (47)	1.4 (0.4)

Initial data set+random SCOP*^a^*	All words	11 294	2.5 (0.9)	0.006 (0.4)
	Over-represented words	45 (7)	10.7 (11.9)	1.9 (2.2)

We report average values with standard deviation between brackets. *^a^*: twelve random SCOP classifications were generated by permuting the loops in the real SCOP classification.

### Link between extreme ubiquitous words and known structural motifs

We focused on extreme ubiquitous words, defined by *Lp_max _*≥= 10 *nb*_sf* _≥= 5. As reported in Table 2 these 24 words account for only 0.2% of words but cover more 5% of loop-length and are seen in 63% of proteins (see Figure 4 for the definition of coverages). These words are highly recurrent, with a mean occurrence equal to 326 (± 216). They are seen in 32 to 285 superfamilies and over-represented in 5 to 25 superfamilies.

Some recurrent structural motifs in loops are well characterized and described in the literature. These motifs include *β*-turns [54,55], *α*-turns [56] and *γ*-turns [57,58], nests [49] and niches [50]. They may play a role in protein folding and stability [59,60] or in the biological function of proteins, within the enzyme active sites or binding sites [49,61]. We thus investigated whether extreme ubiquitous words correspond to some of these small structural motifs. The results of this analysis are reported in Table 4.

**Table 4:** Correspondence between extreme ubiquitous words and small structural motifs

	Statistics in the initial data set			Comparison with known motifs		
**Word**	**Occurrence**	***Lp_max_***	*nb*_sf*_/*nb_sf_^a^*	**Known motif**	**Match*^b^***	**Precision (%)**

				*β*-turn comparison		

PZCD	903	34.82	25/211	*β*-turn	902	100
HBDS	1588	21.97	22/285	*β*-turn	1588	100
ZCDS	1112	27.55	22/246	*β*-turn	996	88
UFQK	449	27.77	15/134	*β*-turn	441	98
GYUQ	278	14.40	11/96	*β*-turn	278	100
YBDS	391	20.60	9/136	*β*-turn	391	100
FQLG	242	25.37	8/77	*β*-turn	236	98
YZDS	397	10.30	7/130	*β*-turn	394	99
GUDO	43	27.55	6/11	*β*-turn	43	100
FFFI	265	21.62	6/80	*β*-turn	206	78
FQKG	237	32.77	5/71	*β*-turn	223	94

				Motivated Proteins comparison*^c^*		

SLGI	258	15.60	8/114	*β*-turn end	11 (13)	85
QLGI	185	15.16	7/89	*β*-turn end	4 (4)	100

DRPI	232	14.95	14/94	Nest	9 (10)	90
DSPI	541	27.15	9/158	Nest	14 (15)	93
DSGI	387	32.45	7/115	Nest	20 (20)	100
DSKG	346	23.16	9/145	Nest	9 (9)	100
DSKH	411	20.46	7/145	Nest	10 (10)	100
DOIP	219	63.30	7/82	Nest	10 (10)	100
OIPI	201	69.81	8/71	Nest	11 (11)	100

HBBQ	616	23.29	10/219	Niche	23 (23)	100
BQGI	337	21.06	9/130	Niche	18 (19)	95

SKGI	34	18.93	12/127	-	NA	
DGPI	56	15.77	5/32	-	NA	

*^a^*: *nb*_*sf *_denote the number of SCOP superfamilies in which a structural word occurs. *^b^*: match denotes the number of fragments containing a known motif. *^c^*: comparison with Motivated Proteins motifs is restricted to the set of proteins common to our database and the Motivated Proteins database. In this case, the number between brackets denotes the number of fragments involved in the comparison.

#### β-turn motifs

We compared extreme ubiquitous words and standard *β*-turns [54,55]. As *β*-turns are four-residue long and we consider seven-residue motifs, the question is to know whether *β*-turns are included in, or overlap with extreme ubiquitous words. As shown in Table 4, eleven structural words (PZCD, HBDS, ZCDS, UFQK, GYUQ, YBDS, FQLG, YZDS, GUDO, FFFI, FQKG) are clearly associated with *β*-turns, and two words (SLGI, QLGI) contain the three last residues of a turn motif. To evaluate the structural diversity of this set of eleven extreme ubiquitous words, we computed the *α*-carbon Root-Mean-Square Deviation (RMSD) between all word-pairs. The RMSD between two words is measured by the average RMSD between 30 fragment pairs randomly selected within pairs of seven-residue fragments encoded by the two words. The set of eleven words clearly associated with *β*-turns comprises structural words with very different conformations, with a mean RMSD of 2.12 Å (± 1.05). This reflects the diversity of *β*-turns motifs. For example, word PZCD contains two type I turns, whereas word UFQK contains one type II turn.

**Figure 6:**
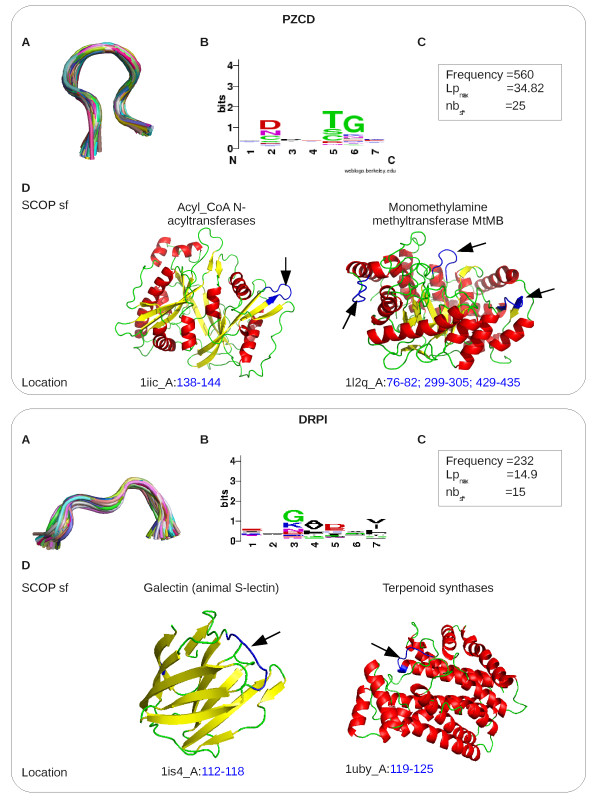
**Illustration of two ubiquitous structural words**. Upper part: structural word PZCD. Lower part: structural word DRPI. A: geometry of several word fragments, optimally superimposed. B: amino-acid conservation of the word generated by WebLogo http://weblogo.berkeley.edu/. C: word statistics. D: example of structures containing the structural word. The location of structural word is indicated by arrows.

An example of an extreme ubiquitous structural word corresponding to *β*-turn motifs, word PZCD, is illustrated in Figure 6 (upper panel). The superimposition of PZCD-fragments and the amino-acid logo [62] associated to the PZCD-fragments, presented in Figure 6A and 6B, shows that PZCD-fragments are very similar in terms of structure and present some amino-acid specificities at positions 2, 5 and 6. As shown in Figure 6C, this word is very frequent (seen 560 times in the initial data set), and over-represented in 25 superfamilies with an *Lp_max _*equal to 34.82. The representation of two proteins containing PZCD-fragments shows that this ubiquitous word is present in superfamilies with different folds. As reported in Table 4, 99.8% of PZCD-fragments contain *β*-turns. Specifically, they contain two *β*-turns, at positions 2:5 and 3:6.

 However, some of the fragments encoded by the eleven words strongly associated with *β*-turns, given in Table 4, do not contain turns as assigned by the ExtractTurn software. This represents a small fraction of the fragments: only 342 fragments out of 8 369, i.e. 4%. Out of these 342 fragments, 79 fail the turn assignment because they have a  distance greater than 7 Å and 263 because they have an internal residue in the helical state. For example, only one of YZDS-fragments is not identified as a turn because the distance is equal to 7.08 Å (2ahu_A: 259-262). Our structural words therefore group together fragments including fragments identified as turns and some that narrowly fail the turn assignment. This suggests that structural motifs could be used to assign "relaxed" turns and supports the notion of turn-like conformations, introduced by *Fuchs et al*, corresponding to four-residue fragments with a  distance around 7 Å [63].

#### Nest or niche motifs

We also compare extreme ubiquitous words with the 12 small hydrogen-bonded 3D motifs extracted from the Motivated Protein database [48]. Results of this analysis are reported in Table 4. As stated in the Methods section, there is very little overlap between our initial data set and the proteins stored in the Motivated Protein database. Even on such a small number of fragments, the comparison reveals that seven extreme ubiquitous words (DRPI, DSPI, DSGI, DSKG, DSKH, DOIP and OIPI) correspond to nest motifs, with precision greater than 93% and two words (BQGI and HBBQ) correspond to niche motifs with precision greater than 95% precision. The set of words corresponding to nest motifs includes structural words with similar conformations, such as DRPI, DSPI and DSGI or DSKG and DSKH. We also note that some structural words overlap: in 81% of cases, structural word DOIP is immediately followed by letter I, forming the five-structural letter word DOIPI.

Figure 6 (lower panel) provides an example of a structural word, DRPI, containing a nest motif. We observe that DRPI-fragments are very similar in terms of structure and present some weak amino-acid specificities in positions 3: 5 and 7. This word is recurrent (seen 232 times in the initial data set and in 94 superfamilies) and over-represented in 15 superfamilies with a *Lp_max _*equal to 14.9. The representation of two proteins containing the DRPI word shows it is present in superfamilies with different folds.

Like turn motifs, nest and niche motifs are detected by applying geometrical thresholds. In this case also, the fact that a very small proportion of our fragments fail the assignment suggest that structural words could be used to assign nest- and niche-like motifs.

#### Extreme ubiquitous words not associated to known structural motifs

Two ubiquitous words, DGPI and SKGI, are extracted from proteins not listed in the Motivated Protein database. It is therefore not possible to compare them with niche and nest motifs. Let us note, however, that DGPI is structurally close to the structural word DRPI (RMSD equal to 0.74 ± 0.24 Å), which contains nest motifs. In the same way, SKGI is similar to SLGI (RMSD equal to 0.76 ± 0.24 Å), a word containing the end of a *β*-turn.

### Link between ubiquitous words and functional annotations

In the previous part, we have shown that extreme ubiquitous words contain some known motifs such as turns, nest, niche. It has been shown that these small motifs could be involved in protein functions such as active sites or binding sites [49,61]. We thus surveyed the association between extreme ubiquitous words and Swiss-Prot annotation by computing the precision of the extreme ubiquitous words toward biological annotations. As reported in Additional file 1: Table S1, we obtained low precisions, suggesting that ubiquitous words are not strongly associated to functional features.

### Link between extreme superfamily-specific words and biological annotations

Unlike ubiquitous words, superfamily-specific words are highly over-represented in few superfamilies, suggesting a possible implication in function. In this section, we focus our analysis on the extreme superfamily-specific words, defined by *Lp_max _*≥= 50 and *nb*_sf* _< 5, and investigate their correspondence with biological annotations provided by Swiss-Prot extracted from the annotation data set. We complement the analysis based on Swiss-Prot by the use of external softwares (Rep, SitePredict, CSA and LigPlot) for functional site identification/prediction.

As reported in Table 2, extreme superfamily-specific words account for 0.2% of the structural words, 0.7% of the seven-residue fragments, and are seen in 17% of the proteins of the initial data set. Their average *Lp_max _*score is equal to 88.9 ± 46, ranging from 51.7 to 210, and their mean *nb*_sf* _is equal to 1.4 ± 0.4. The results of the comparison between extreme superfamily-specific words and Swiss-Prot annotations are reported in Table 5. We present below these results grouped according to the Swiss-Prot annotations identified during the comparison. For each annotation, we computed the precision, i.e. fraction of the fragments encoded by a structural word that actually correspond to the annotation. A structural word associated to a precision greater than 40% with respect to a functional annotation is said to be *functional*. For these functional words, we also computed the sensitivity, i.e. fraction of the annotation that is actually covered by the structural word.

**Table 5:** Correspondence between extreme superfamily-specific words and Swiss-Prot annotations in the initial data set

			Statistics in the initial dataset		Comparison with Swiss-Prot		
**Word**	**Occ*^a^***	***Lp_max_***	*nb*_sf*_/*nb_sf_^b^*	**Superfamilies*^c^***	**Annot**	**Match/total (Precision (%)) *^d^***	**Sensitivity (%)**

**URNH**	43	54.95	1/17	48726*	Disulfide	7/14 (50)	4
**RNHB**	59	51.33	1/28	48726*	Disulfide	9/20 (45)	6

**UQHS**	53	75.07	1/16	52058*	Repeat	12/22 (55)	41
**SUQH**	70	63.42	1/25	52058*	Repeat	11/26 (42)	38
**QHSG**	37	51.75	1/12	52058*	Repeat	4/10 (40)	14
**HSGI**	63	76.26	1/18	52058*	Repeat	5/12 (42)	17
*QXUS*	43	52.05	1/10	51735*	Repeat	1/15 (7)	
*ZSGI*	99	52.22	1/49	52058*	Repeat	7/36 (19)	
*GSUS*	169	140.49	3/59	141571*, 52047, 52058	Repeat	6/38 (16)	
*GZDO*	115	84.72	3/49	47473*, 52833, 52935	Repeat	1/35 (3)	

**DODQ**	73	157.01	1/17	47473*	CA_BIND	15/23 (65)	75
**ZDOD**	48	91.27	1/13	47473*	CA_BIND	11/16 (69)	58

**YUOD**	111	184.67	1/11	52540*	NP_BIND	39/41(95)	35
**UODO**	142	210.14	4/14	52540*,53659, 54211, 55729	NP_BIND	49/60 (82)	38
**OEIJ**	33	53.84	1/4	51735*	NP_BIND	6/7 (86)	14
**EIJU**	48	51.68	1/13	51735*	NP_BIND	7/15 (47)	20
*USLG*	121	137.35	2/47	141571*, 51206	NP_BIND	2/22 (9)	
*UZCI*	99	63.70	2/28	103025*, 56784	NP_BIND	1/13 (8)	

**RUDO**	27	55.55	1/4	53335*	Binding	5/10 (50)	18

*UGRU*	37	60.07	1/8	53335*	Binding	4/12 (33)	

*EGZD*	48	51.68	1/5	51735*			
*GRUD*	33	70.55	1/6	53335*			
*SLGS*	60	118.45	1/17	141571*			

This comparison is made on a subset on the initial set: 1487 proteins that can be mapped to biological annotations using the PDB/UniProt Mapping database. *^a^*: word occurrence. *^b^*: *nb_sf _*denotes the number of SCOP superfamilies in which the structural word is seen. *^c^*: superfamilies in which the word is over-represented. *^d^*: match and total denote the number of fragments annotated and the total number of fragments, respectively. Bold font indicates a match/total ratio greater than 40%. Italic font indicates a match/total ratio lower than 40%. Abbreviations used: NP BIND = nucleotide phosphate-binding site, CA_BIND = calcium-binding site. SCOP ids: 103025 = Folate-binding domain, 141571 = Pentapeptide repeat-like, 47473 = EF-hand, 48726 = Immunoglobulin, 51206 = cAMP-binding domain-like, 51735 = NAD(P)-binding Rossmann-fold domains, 52047 = RNI-like, 52058 = L domain-like, 52540 = P-loop-containing nucleoside triphosphate hydrolases, 52833 = Thioredoxin-like, 52935 = PK C-terminal domain-like, 53335 = S-adenosyl-L-methionine-dependent methyltransferases, 53659 = Isocitrate/isopropylmalate dehydrogenase-like, 54211 = Ribosomal protein S5 domain 2-like, 55729 = Acyl-CoA N-acyltransferases (Nat), 56784 = HAD-like. "*" denotes the superfamily in which the word is most over-represented.

#### Disulfide annotation

Two overlapping extreme superfamily-specific words, RNHB and URNH, are strongly over-represented in the immunoglobulin superfamily (SCOP id = 48726). They correspond to regions covalently linked by disulfide bridges and identified by the "Disulfide bond" Swiss-Prot annotation with a precision of 50 and 45%. This annotation provides no functional information *per se*, but might indicate that these structural motifs result from structural constraints induced by the disulfide bridge. However, the very low sensitivity observed (4 and 6%) shows that a only small fraction of the disulfide annotations are encoded by these words.

#### Repeat annotation

Four overlapping extreme superfamily-specific words SUQH, UQHS, QHSG, HSGI are strongly over-represented in the "L domain-like" superfamily (SCOP id = 52058). This superfamily groups proteins containing repeat regions, which are regions of 20 to 30 amino acids unusually rich in leucine [64]. Repeat regions have strong implications for the biological role of protein, as they are often involved in protein-protein interactions in plant and mammalian immune responses [64]. A number of human diseases have been shown to be associated with mutations affecting leucine-rich repeat domains [64]. These repeat regions may therefore be of functional relevance.

**Figure 7:**
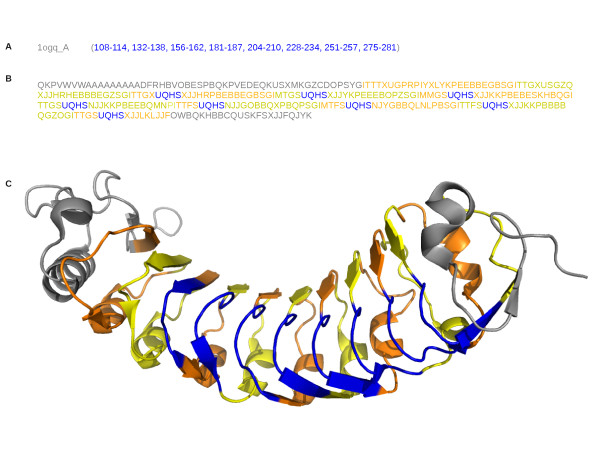
**Illustration of the word **UQHS** corresponding to the repeat annotation**. A: position of UQHS word in protein 1ogq A. B: structural-letter sequence of the protein 1ogq_ A. C: representation of the 3D structure of this protein. Blue: UQHS-fragments. Orange: odd-numbered repeat regions. Yellow: even-numbered repeat regions.

Structural words SUQH, UQHS, QHSG,HSGI often occur in the same proteins, allowing the formation of longer motifs, like illustrated in Figure 7: in protein 1ogq A, SUQH and UQHS overlap to form the five-structural letter words SUQHS.

**Figure 8:**
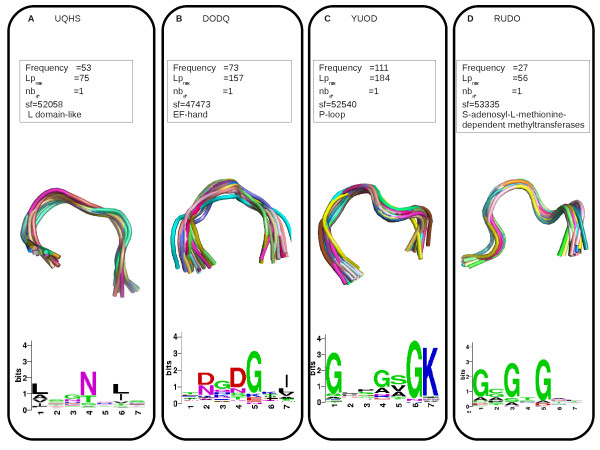
**Illustration of four functional words**. A: structural word UQHS. B: structural word DODQ. C: structural word YUOD. D: structural word RUDO. For each word, we provide word statistics (frequency, *Lp_max_*, *nb*_sf*_), the name of the superfamily in which the word has highest *Lp *score, the superimposition of fragments associated with this word, and amino-acid conservation data.

Figure 8A illustrates the example of the word UQHS. It is a recurrent word (seen 52 times in the initial data set), strongly over-represented in one superfamily (SCOP id = 52058), with a high maximal score (*Lp_max _*= 75.07). The superimposition UQHS-fragments shows that they are very similar in terms of structures, with a turn conformation. The amino-acid logo indicates that UQHS presents amino-acid conservation at positions 1, 4 and 6, resulting in an amino-acid profile close to the consensus sequence of LRR (LxxLxLxxNxL or LxxLxLxxCxxL [65]).

The comparison with Swiss-Prot annotations reveals that the four structural words SUQH, UQHS, QHSG and HSGI correspond to the "repeat" annotation with precision greater than 40% (see Table 5). According to our definition of functional words, these four words are thus functional. Some fragments encoded by these functional words, however, do not correspond to repeat annotations. For example, in the initial data set, 10 UQHS-fragments are unannotated. To determine whether these 10 fragments might still correspond to repeat regions unannotated in Swiss-Prot database (i.e., false negatives), we used the REP software to predict repeat regions. Two repeat regions are predicted: 1dce A:484-507 and 529-553. Region 1dce A: 484-507 actually contains the word UQHS, whereas the second region: 529-553 does not (see Table S2). 

The sensitivity measure for the repeat annotation for the four structural words SUQH, UQHS, QHSG and HSGI ranges from 17 to 41%, meaning that repeat regions correspond to a variety of conformations, not only the ones encoded by SUQH, UQHS, QHSG and HSGI. By definition, repeat regions are formed by the repetition of a motif.

#### Calcium-binding site annotation

Two overlapping extreme superfamily-specific words, ZDOD and DODQ, are over-represented in only one superfamily: "EF-hand" (SCOP id = 47473). This superfamily contains proteins with EF-hand units, which consist of two helices connected by a calcium-binding loop. The words ZDOD and DODQ are frequently overlapping: in 66% of cases, DODQ is preceded by the letter Z, forming the word ZDODQ. Figure 8B presents the statistics, geometry and amino-acid sequence conservation of the word DODQ. The amino-acid logo shows that DODQ presents amino-acid conservation at positions 2, 3, 4, 5 and 7, with a strong conservation of an aspartic acid or asparagine residue at positions 2 and 4 and of a glycine residue at position 5. This conserved sequence is in close agreement with the consensus sequence of calcium-binding motifs [DxDxDG] [66].

**Figure 9:**
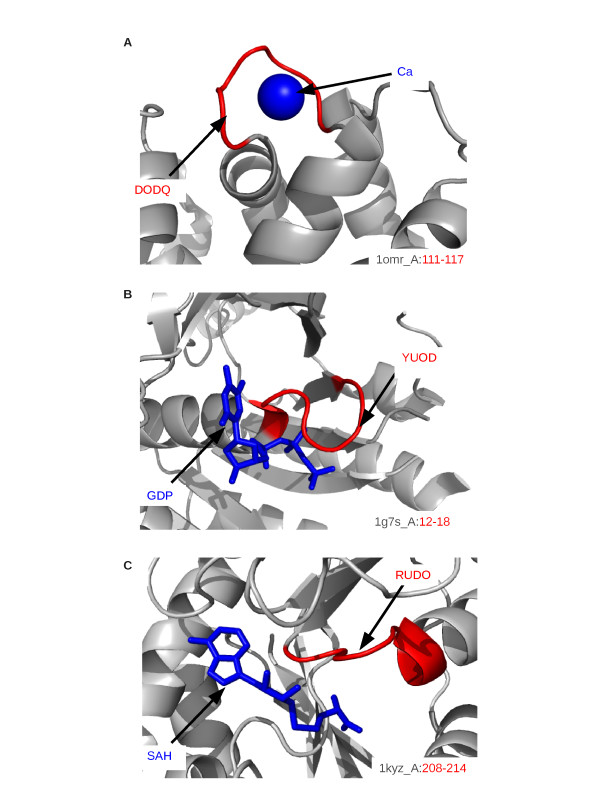
**Illustration of the functional role of three words**. A: DODQ corresponds to calcium-binding sites. B: YUOD contains residues involved in nucleotide-binding sites. C: RUDO contains residues involved in SAH/SAM-binding sites. Structural words are highlighted in red and ligands in blue.

The two words ZDOD and DODQ correspond to the calcium-binding site annotation (CA_BIND) with precision greater than 65%, they thus are functional motifs. As shown in Figure 9A, DODQ contains residues directly involved in the binding of calcium ions. Five ZDOD-fragments and nine DODQ-fragments are not annotated as calcium-binding sites in Swiss-Prot. However, six of these unannotated DODQ-fragments are identified as putative calcium-binding sites by the SitePredict software (see Table S3). The sensitivity of the calcium-binding site annotations with respect to ZDOD and DODQ ranges from 58 to 75%, meaning that the majority of calcium-binding sites actually correspond to these structural words. These two structural words could thus be used to predict calcium-binding site candidates.

#### Nucleotide-binding site annotation

Five extreme superfamily-specific words are associated with nucleotide-binding site annotations (NP_BIND) with precision greater than 47%. Some correspond to ATP/GTP-binding sites, others to NAD(P)-binding sites. We discuss these two cases separately.

**ATP/GTP-binding sites **Structural words YUOD and UODO are strongly over-represented in the superfamily "P-loop-containing nucleotide triphosphate hydrolase" (SCOP id = 52540), grouping proteins with a phosphate-binding site. These two words are often found in the same proteins: in 90% of cases, the structural word YUOD is followed by the letter O, forming the word YUODO.

Figure 8C illustrates the statistics, geometry and amino-acid sequence conservation of the YUOD word. This word displays clear amino-acid conservation: glycine in positions 1 and 6, lysine in position 7, and threonine or serine in position 8, consistent with the consensus sequence of P-loops: [AG]XXXXGK[TS] [10].

Structural words YUOD and UODO correspond to the nucleotide-binding site annotation with precision greater than 80%. YUOD and UODO are thus functional words with residues directly involved in ATP/GTP-binding sites, as shown in Figure 9B for YUOD word. In the initial data set, two YUOD-fragments and eleven UODO-fragments are unannotated. SitePredict indeed predicts ATP/GTP-binding sites for four of the eleven unannotated UODO-fragments (see Table S4). The sensitivity is equal to 35 and 38%, meaning that roughly one third of the ATP/GTP-binding sites adopt conformations described by these structural words.

**NAD(P)-binding sites **Two structural words, OEIJ and EIJU are strongly over-represented in the "NAD(P)-binding Rossmann-fold domain" superfamily (SCOP id = 51735) grouping proteins with NAD(P)-binding sites. These words are often overlapping: in 95% of cases, OEIJ is followed by the letter U.

Word OEIJ is associated with the NP_BIND annotation with precision equal to 86% and 47% respectively, they thus are functional words. One OEIJ-fragment and seven EIJU-fragments are unannotated. Two of the seven unannotated EIJU-fragments are predicted as NAD(P)-binding sites by SitePredict (see Table S5). The sensitivity is quite low, ranging from 14 to 20%, meaning that NAD(P)-binding sites probably adopt various conformations, and not only the ones encoded by OEIJ and EIJU.

#### S-adenosyl-L-methionine binding sites

The superfamily-specific word RUDO is strongly over-represented in the "S-adenosyl-L-methionine-dependent methyltransferase" superfamily (SCOP id = 53335), grouping proteins with SAH/SAM-binding sites. Figure 8D presents the geometry of the structural word RUDO and its amino-acid signature, with glycine residues preferred at positions 1, 3 and 5. Figure 9C presents an illustration of a SAH/SAM-binding site for a RUDO-fragment, showing the residues involved in the SAH/SAM-binding site. This word corresponds to the "binding" annotation with a precision equal to 50%, therefore it is a functional word. Three out of the five unannotated RUDO-fragments actually correspond to SAH/SAM-binding sites according to our analysis using LigPlot. The sensitivity is equal to 18%, suggesting that SAH/SAM-binding sites adopt other conformations than the one identified by the RUDO word.

#### Unannotated extreme superfamily-specific words

Ten superfamily-specific structural words QXUS, ZSGI, GSUS, GZDO, USLG, UZCI, UGRU, EGZD, GRUD and SLGS, indicated in italics in Table 5 could not be validated as functional motifs because they have low precision values toward Swiss-Prot annotations. This could be due to (i) the limited number of proteins of the initial data set that are annotated in Swiss-Prot and (ii) the incomplete annotation of Swiss-Prot, since annotations for a given protein simply reflect our current knowledge about it.

### Double checking the link between functional words and biological annotations using the validation data set

The previous analysis was based on the Swiss-Prot annotations of the annotation data set. Since many proteins of the initial data set are lost in the UniProt/PDB mapping step, we complement our results using a data set specifically built to maximize the coverage by Swiss-Prot: the validation data set composed of 2 636 proteins. In the validation data set, 17% of seven-residue fragments in loops are covered by a Swiss-Prot annotation versus only 2% in the initial data set.

For the functional words identified in the previous section, we compute the precision and sensitivity measures presented in Table 6. We do not consider the words associated to disulfide and the repeat annotations since they are non specific to annotations. The seven functional words considered have precision greater than 40%, the threshold used for their validation in the annotation data set. These two criteria are stable on the annotation and validation sets with sligth global increase for the validation set: on average 70% to 76% for precision and 37% to 39% for sensitivity. The precision values are high indicating that most of the fragments encoded by these words are annotated by the corresponding annotation.

**Table 6:** Precision and sensitivity for functional words computed in the validation data set

Words	Annotation	Second-level annotation	Precision (%)	Sensitivity(%)
DODQ	CA_BIND	-	82	95
ZDOD	CA_BIND	-	92	64

YUOD	NP_BIND	ATP/GTP	91	29
UODO	NP_BIND	ATP/GTP	80	40
OEIJ	NP_BIND	NAD(P)	94	7
EIJU	NP_BIND	NAD(P)	54	10

RUDO	Binding	SAH/SAM	44	30

## Discussion

In this work, we used a structural alphabet-based simplification of protein structures and applied an exact statistical approach to identify structural motifs over-represented in loops in SCOP superfamilies. Our underlying hypothesis was that structural words with unexpectedly high frequency are probably linked to structural or functional implication. We discovered two distinct trends: some words, termed ubiquitous words, are over-represented in several superfamilies, whereas others, termed superfamily-specific words, are over-represented in a small number of superfamilies. We then investigated the link between these structural motifs and known structural motifs and functional sites annotated in Swiss-Prot, on a subset of structural words with extreme over-representation scores.

We focused on structural motifs formed by seven consecutive residues, i.e. four structural letters, since it is the optimal length to have a good description of the 3D conformations and enough data to allow statistical treatments [39]. However, our findings revealed longer motifs formed by overlapping four-structural letter words, such as YUODO, ZDODQ, corresponding to eight-residue motifs or shorter motifs consensus as LGI common to SLGI, QLGI. These results suggest that this motif approach could be extended to motifs of different lengths.

### Interpretation of ubiquitous words

Since ubiquitous words are over-represented in several SCOP superfamilies with various functions, it is likely that they are the result of structural rather than functional requirement. A comparison of ubiquitous words with extreme scores and known small 3D motifs showed that extreme ubiquitous words contain *β*-turn, nest or niche motifs. Several studies have shown that turns, nest and niche motifs may play a functional role in determining the conformation of enzyme active sites and binding sites [13,49,61]. We were not able to confirm this point using our extreme ubiquitous words. However, among the functional words identified in the subset of extreme superfamily-specific words, three words (ZDOD, UQHS, UODO) actually contain turns, which is in agreement with the fact that turn motifs could be involved in binding sites [13]. Let us note that turns, niches and nests are shorter (three or four residues) than our structural words (seven residues). The fact that we capture them using structural words suggests that structural motifs longer than previously described are important for protein folding and stability. Long structural motifs are thus part of a "basic structural repertoire", similarly to regular secondary structures which are used in protein structures regardless of the overall fold and function of the protein concerned. In addition, structural words allow detecting structural motifs without computing hydrogen bonds, or dihedral angles, and without explicit pairwise comparison of fragments. This could thus be very useful to detect structural motifs with relaxed parameters like turn-like motifs.

### Interpretation of superfamily-specific words and their link with function

#### Usage of superfamily-specific words for functional site prediction

The analysis of the correspondence between extreme superfamily-specific words and Swiss-Prot annotations revealed that some of superfamily-specific words are linked to functional sites. For example, we found superfamily-specific words associated to repeat annotations and binding sites to ATP/GTP, SAM/SAH, NAD(P), calcium and iron. Thus functional words allow a reliable prediction of some binding sites.

#### Limitations introduced by the Swiss-Prot mapping

Some annotations, such as metal-binding sites (cadmium, lithium, mercury, potassium, vanadium) are very rare and not represented in our data set. This explains why these functional sites are not detected at all by superfamily-specific words. Moreover, only a fraction of the annotation data set is covered by Swiss-Prot annotations (2% of seven-residue fragments) and the step of mapping annotations to PDB structures using the PDB/UniProt Mapping database further reduces significantly the data available for comparison. The link between structural words and functional sites is thus established on a limited amount of data and is probably under-estimated by our analysis. For example the structural word UGRU, over-represented in the "S-adenosyl-L-methionine-dependent methyltransferase" superfamily (SCOP id = 53335), is not characterized as "functional word" in the annotation or validation data sets (precision = 33% and 36%). The manual analysis of the functional annotations of UGRU-fragments show that 69% of them are actually involved in SAH/SAM-binding sites, see Table S6. This illustrates the case of a functional motif missed by our analysis due to a defect of biological annotations.

In this paper, the link between superfamily-specific words and functional sites is established only for the 23 extreme superfamily-specific words. These 23 words cover 1% of residues in loops and they are seen in 17% of proteins. If we consider superfamily-specific words with moderate scores (565 words with *Lp_max _*≥ = 10, see Table 2), the coverage can be increased to 10% of residues and 90% of proteins. From these moderately superfamily-specific words, 13 words are clearly associated with a functional Swiss-Prot annotation ("binding site" or "active site" annotations), 17 correspond to a repeat annotation and 16 to a disulfide annotation (data not shown). For example, word ZCLH is over-represented in the superfamily SCOP id = 53474 with a *Lp_max _*equal to 12. This word has a precision for the detection of "active site" annotation of 67% (see Table S7). This suggests that over-represented words with moderate *Lp_max _*score may be functional too.

#### Intrinsic limitation of the structural word approach

However, some functional sites were not detected by structural words. To be identified by our structural word approach, a functional site must meet two conditions: (i) at least one part of the functional site must be located in protein loops and (ii) it must correspond to recurrent structures across different proteins. Indeed, structural words can only identify a functional motif if structural conformation spanning at least seven or more consecutive residues. Thus, superfamily-specific words cannot detect DNA-binding sites or zinc finger motifs because these functional sites are preferentially seen in α-helices. In the same way, some metal binding sites (cobalt, copper, magnesium, canganese, colybdenum, nickel, sodium) are not detected because they display a high flexibility [67] or a structural conservation restricted to few residues.

**Figure 10:**
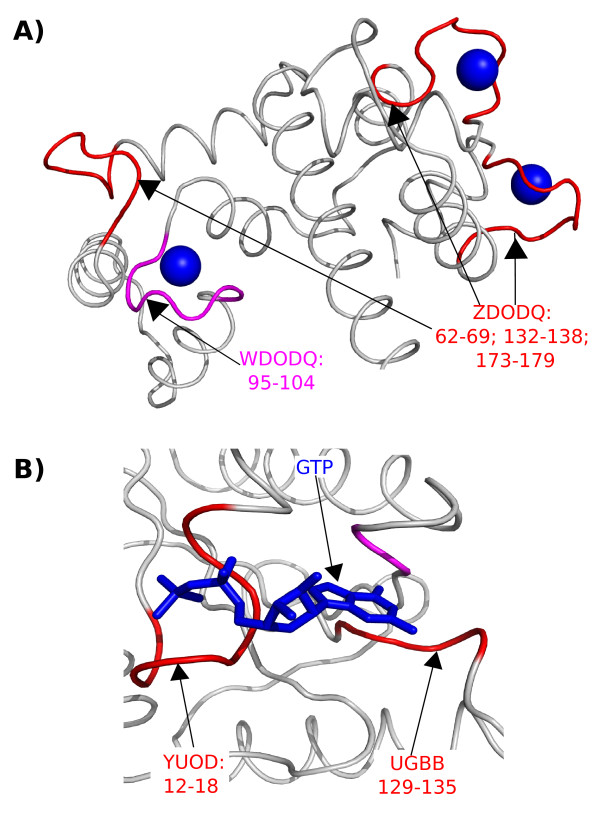
**llustration of the binding sites, which correspond to different words**. A: Illustration of the flexibility of calcium-binding sites in the Calcium-dependent protein kinase 3 (pdb code 3k21), which is cristallized with 3 calcium atoms (colored in blue). Among these 3 calcium-binding sites two are detected by overlapping words ZDOD and DODQ, colored in red. The third binding site is detected by overlapping words WDOD and DODQ, colored in magenta. B: Illustration of a GTP-binding site involving different 3D regions in the Translation initiation factor if2/eif5b (pdb code 1g7s). The GTP is represented in blue. The binding site is composed of three 3D regions (15-20, 130-133; 198-199). In red are colored the two regions, which are detected by superfamily-specific words: YUOD and UGBB over-represented in the superfamily "P-loop containing nucleoside triphosphate hydrolases" (52540). In magenta is colored the third region, which is not detected by superfamily-specific word. In Swiss-Prot this protein is annotated by two NP_bind annotations (12-19, 76-80, 130-133).

To quantify the correspondence between extreme superfamily-specific words and Swiss-Prot annotations, we computed the precision and sensitivity of annotation detection by these words. We observed that sensitivity values depend on the functional sites and structural words. For example, two overlapping words DODQ, ZDOD present a high sensitivity for calcium-binding sites, meaning that most of these binding sites can be detected by these two structural words. Other structural words have lower sensitivity, e. g. YUOD detects only one third of ATP/GTP-binding sites. However, we checked, on randomized data sets, that these sensitivity measures are significantly greater than expected by chance (see Table S8). Indeed, random sensitivities are very low and the sensitivity of structural words reported in this study are higher in any case. Thus, even if the sensivity measures reported in this sudy may seem modest, they are still significant, meaning that all the superfamily-specific structural words presented here are significantly enriched in functional sites. These low sensitivity values indicate that some functional sites actually correspond to several conformations encoded by different structural words. These different conformations of a functional site could be explained by (i) its flexibility or (ii) the fact that it can span several segments in a protein. Figure 10 presents an illustration of flexibility of binding-site through the four calcium-binding sites of protein Calcium-dependent protein kinase 3 (pdb code 3k21). This flexibility results in the encoding of these functional sites into two close words: ZDOD and WDOD, with a RMSD of 0.419 Å. A way to take into account the flexibility of binding-site could be to consider "degenerated words" (for example [W/Z]DOD) instead of "exact" word. This would certainly increase the ability to detect functional sites.

In Figure 10, we also present an example of protein Translation initiation factor if2/eif5b (pdb code 1g7s) data, illustrating a binding site involving different 3D regions. This protein contains a GTP-binding site involving three regions, which two are annotated by one NP_BIND annotation, resulting in two NP_BIND annotations for this protein. Each annotated region is detected by a superfamily-specific word: YUOD and UGBB. This indicates each word can detect one part of the GTP-binding site, thus each word is expected to detect to 50% of the NP_BIND annotations at most. Thus, the weak sensitivity value of some functional words shows that these words can detect one part of the functional site. To identify the entire functional sites, we could couple the different functional words associated to the same annotation.

#### Comparison with existing approaches

Several approaches address the link between local structures and protein function. These methods can be clustered into three groups.

The first group corresponds to the characterization of structural motifs specific to functional sites [22-28]. Such methods consist in learning the structural motifs of known functional sites and are therefore dedicated to the prediction of those sites.

The second group corresponds to the discovery of conserved structural motifs in proteins with the same function. These methods start from protein superfamilies and search for structural motifs specific to superfamilies [20,21,68]. They can identify conserved motifs in different proteins with the same function. In these approaches, the extraction of structural motifs is based on the comparison of structural fragments using RMSD. These methods are able to discover new functional sites within superfamilies. However, they cannot identify functional motifs common to several superfamilies.

The third group corresponds to structural classification of local conformations, followed by an analysis of the association between clusters and functional sites [14,17,18,69]. These methods do not focus on the description of a particular functional site, or restrict the analysis to a particular superfamily. Instead, they analyze *a posteriori *the association between fragment clusters and protein superfamilies or GO annotations. Our approach is based on the same philosophy as these methods.

Compared to Espadaler et al. [14], Tendulkar et al. [17], and Manikandan et al. [18], our method is original in three ways: (i) the extraction of structural motifs is based on a structural alphabet, which allows defining structural motifs without using geometrical thresholds or extensive pairwise structural comparison, (ii) the functional role of a motif in a particular superfamily is assessed by its statistical over-representation within the superfamily, and (iii) it can deal with all loops, irrespective of their length or secondary structure types. This last point is particularly important: in a previous study, we have shown that 64% of structural words display no specificity for loop length [39]. It is also the case of the functional motifs identified in the present study: for example, 60% fragments of the word DODQ, involved in calcium-binding sites are extracted from short loops, and 40% from long loops. The fact that we made a systematic decomposition of loops into structural words, instead of clustering full-length loops as done by Espadaler *et al*. [14] makes the comparison with their study difficult.

Two studies by Tendulkar *et al*. [17] and Manikandan *et al*. [18] aimed at the extraction of structural motifs specific to a protein function. Contrary to our approach, they considered all structural motifs including *α*-helices and *β*-strands. In these two studies, structural motifs were extracted by a systematic classification of eight-residue fragments based on geometric invariants [17] or dihedral angles [18]. They then analyzed the association between structural clusters and protein functions provided by SCOP superfamilies [17] or GO terms [18]. Tendulkar et *al*. [17] defined a cluster as functional if at least 70% of its fragments are found in a same SCOP superfamily. Manikandan et *al*. [18] identified functional clusters on the basis of the over-representation of GO terms in clusters. These two definitions restrict the definition of functional motifs to motifs specific of one superfamily or GO term. By contrast, the statistical treatment presented here allows the extraction of motifs shared by several families, even if the superfamily contains few members.

Recently, Wu *et al*. [69] have proposed an approach to extract functional structural motifs from DNA-binding proteins using a structural alphabet. As in our approach, the structural alphabet is used to simplify 3D structures into uni-dimensional sequences. The structural alphabet used in [69] is composed of 16 structural letters, named protein blocks. Wu *et al*. focused on DNA-binding sites by searching structural words present in DNA-binding proteins binding and absent in others, and considered long and degenerated structural words (26 residues) without secondary structure restriction. In the present study, we discarded helices and strands. In addition, our statistical treatment is radically different from theirs, and allows retrieving structural words shared by several superfamilies, even in superfamilies with few proteins. Even if based on a similar method of protein structure simplification, both these works thus pursue quite different objectives and consider different structural motifs.

## Conclusion

In this study, we present a systematic extraction of 3D motifs from loops likely to be important for protein structure or function. This method is based on the structural alphabet HMM-SA and an advanced method for pattern statistics. We identified *ubiquitous *structural motifs over-represented in several superfamilies, and *superfamily-specific *structural motifs over-represented in few superfamilies. Some ubiquitous words correlate with known 3D motifs such as *β*-turns, niches and nests. The link between the word over-representation and functionality was proved for some superfamily-specific words. Thus, some of these structural words allows the detection of calcium-binding sites, some part of nucleotide, SAH-binding sites, or active site. As in DNA sequence analysis, statistical over-representation can be related to functional features.

These results could be used for the prediction of functional sites in protein structures: the identification of these structural motifs in uncharacterized proteins could provide useful clues to protein function in complement to usual methods based on homologous proteins.

As some functional annotations are supported by regular secondary structures, current perspectives include the consideration of regular secondary structures. Also, some functional words present sequence specificity, which opens the perspective to the prediction of these functional motifs from their amino-acid sequence.

## Authors' contributions

LR, JM, and ACC conceptualized the project. LR developed the software, performed the experiments and drafted the paper. JM extensively edited the manuscript. All authors analyzed the experimental results. All authors contributed to writing the paper and approved the final manuscript.

## Supplementary Material

Additional file 1 **Supplementary information**. This file is a pdf file. It contains different information about the comparison between some over-represented words and biological annotations: • Table S1: Precision of annotation dectection by extreme ubiquitous words. • Table S2: Analysis of UQHS fragments. • Table S3: Analysis of DODQ fragments. • Table S4: Analysis of UODO-unannotated fragments. • Table S5: Analysis of EIJU fragments. • Table S6: Analysis of UGRU fragments. • Table S7: Analysis of ZCLH fragments. Table S8 present the results of the computation of a random sensitivity for each functional word. Click here for file
