# Supplementary material for: Dissecting protein loops with a statistical scalpel suggests a functional implication of some structural motifs
Source: BMC Bioinformatics. 2011 Jun 20;12:247. doi: 10.1186/1471-2105-12-247 (PMC3158783; doi:10.1186/1471-2105-12-247)
Supplement: Additional file 1 — Supplementary information. This file is a pdf file. It contains different information about the comparison between some over-represented words and biological annotations: • Table S1: Precision of annotation dectection by extreme ubiquitous words. • Table S2: Analysis of UQHS fragments. • Table S3: Analysis of DODQ fragments. • Table S4: Analysis of UODO-unannotated fragments. • Table S5: Analysis of EIJU fragments. • Table S6: Analysis of UGRU fragments. • Table S7: Analysis of ZCLH fragments. Table S8 present the results of the computation of a random sensitivity for each functional word. [file 1471-2105-12-247-S1.PDF]

Additional Files

Table S1 — Precision of annotation dectection by extreme ubiquitous words

Word	Annotation	Precision
PZCD	DISULFID	6%
HBDS	DISULFID	10%
ZCDS	METAL	8%
UFQK	DISULFID	19%
GYUQ	DISULFID	4%
YBDS	DISULFID	8%
FQLG	DISULFID	5%
YZDS	DISULFID	5%
GUDO	-	0%
FFFI	DISULFID	6%
FQKG	DISULFID	19%
SLGI	REPEAT	5%
QLGI	DISULFID	7%
DRPI	MUTAGEN	1%
DSPI	DISULFID	2%
DSGI	DISULFID	2%
DSKG	DISULFID	8%
DSKH	DISULFID	8%
DOIP	-	0%
OIPI	-	0%
HBBQ	DISULFID	11%
BQGI	DISULFID	4%
SKGI	DISULFID	8%
DGPI	NP_BIND	11%

Table S2 — Analysis of UQHS fragments

Firstly, we compared UQHS fragments with Swiss-Prot annotations. Then, for unannotated fragments, we predicted Repeat regions using REP software (Andrade et al., 2000).
[1]: pdb codes.

Proteins[1]	Word position	Swiss-Prot Annotation	REP
1aor_A	66-72	-	-
1chk_A	84-90	-	-
1dce_A	470-476	-	LRR: 484-507
1dce_A	493-499	-	-
1hye_A	52-58	-	-
1nt4_A	107-113	-	-
1ogq_A	108-114	REPEAT	
1ogq_A	132-138	REPEAT	
1ogq_A	156-162	REPEAT	
1ogq_A	181-187	REPEAT	
1ogq_A	204-210	REPEAT	
1ogq_A	228-234	REPEAT	
1ogq_A	251-257	REPEAT	
1ogq_A	275-281	REPEAT	
1omw_A	554-560	-	-
1ozn_A	185-191	REPEAT	
1ozn_A	209-215	REPEAT	
1ozn_A	233-239	REPEAT	
1ozn_A	64-70	REPEAT	
1vlb_A	175-181	-	-
1w4x_A	436-442	-	-
2cl5_A	198-204	-	-

Table S3 — Analysis of DODQ fragments

Firstly, we compared DODQ fragments with Swiss-Prot annotations. Then, for unannotated fragments, we predicted residues involved in a calcium-binding sites using SitePredict software (Bordner et al., 2008). Only residues predicted with a score higher than 0.5 and matching with studied DODQ-fragments are indicated.

[1]: pdb codes.

Proteins[1]	Word position	Swiss-Prot Annotation	SitePredict
1a12_A	376-382	-	373,382
1dab_A	524-530	-	
1ij5_A	231-237	CA_BIND	
1ij5_A	266-272	CA_BIND	
1ij5_A	333-339	CA_BIND	
1k1x_A	393-399	-	
1k9u_A	14-20	CA_BIND	
1k9u_A	49-55	CA_BIND	
1l1d_A	437-443	-	440,439,441,437
1omr_A	111-117	CA_BIND	
1qls_A	67-73	CA_BIND	
1qus_A	238-244	-	240,242,241,239,243,238,244
1qv1_A	31-37	CA_BIND	
1wdc_B	29-35	CA_BIND	
1wdc_B	98-104	-	-
1wdc_C	95-101	-	98,96,97,100
1xoc_A	347-353	-	349,348,350,351,347,352,353
2h61_A	62-68	-	63,65,64,62,66,67,68,
2pvb_A	52-58	CA_BIND	
2pvb_A	91-97	CA_BIND	
2scp_A	105-111	CA_BIND	
2scp_A	139-145	CA_BIND	
2scp_A	17-23	CA_BIND	

Table S4 — Analysis of UODO-unannotated fragments

Firstly, we compared UODO fragments with Swiss-Prot annotations. Then, for unannotated fragments, we predicted residues involved in a ATP/GTP-binding sites using SitePredict software (Bordner et al., 2008). Only residues predicted with a score higher than 0.5 and matching with studied UODO-fragments are indicated.
[1]: pdb codes.

Proteins[1]	Word position	Swiss-Prot Annotation	SitePredict
1c9k_A	7-13	NP_BIND	
1cnz_A	290-296	NP_BIND	
1cr1_A	313-319	NP_BIND	
1d2n_A	552-558	NP_BIND	
1e2k_A	57-63	NP_BIND	
1e6c_A	10-16	-	-
1f9v_A	475-481	NP_BIND	
1fzq_A	25-31	NP_BIND	
1g6h_A	41-47	NP_BIND	
1g7s_A	13-19	NP_BIND	
1h72_C	90-96	NP_BIND	
1hqs_A	351-357	NP_BIND	
1htw_A	41-47	NP_BIND	
1ihu_A	16-22	NP_BIND	
1ihu_A	335-341	NP_BIND	
1iic_A	177-183	-	177,178,179,180,181,182,183
1in4_A	59-65	NP_BIND	
1jjv_A	10-16	NP_BIND	
1kk1_A	18-24	NP_BIND	
1kkh_A	109-115	NP_BIND	
1ko7_A	152-158	NP_BIND	
1kvk_A	138-144	NP_BIND	
1l2t_A	39-45	NP_BIND	
1l8q_A	120-126	NP_BIND	
1lnz_A	166-172	NP_BIND	
1m0w_A	385-391	-	385,386,387
1mja_A	311-317	-	312,313,314,315,316,317
1mky_A	9-15	NP_BIND	
1mky_A	188-194	NP_BIND	
1nrj_B	46-52	NP_BIND	
1odf_A	39-45	NP_BIND	
1ogo_X	75-81	-	-
1p6x_A	33-39	NP_BIND	
1ptm_A	303-309	-	-
1puj_A	128-134	-	-
1qde_A	66-72	NP_BIND	
1qhx_A	11-17	NP_BIND	
1qsm_A	99-105	-	-
1r2q_A	28-34	NP_BIND	
1svi_A	31-37	NP_BIND	

Continued on next page

			Continued from previous page
Proteins[1]	Word position	Swiss-Prot Annotation	SitePredict
1sxj_A	354-360	NP_BIND	
1sxj_C	54-60	NP_BIND	
1sxj_D	66-72	NP_BIND	
1sxj_E	44-50	NP_BIND	
1u0j_A	335-341	NP_BIND	
1u0l_A	171-177	NP_BIND	
1w0d_A	276-282	NP_BIND	
1w0m_A	145-151	-	-
1w1w_A	34-40	NP_BIND	
1w7j_A	164-170	NP_BIND	
1ye8_A	8-14	NP_BIND	
1z2a_A	17-23	NP_BIND	
2bcg_Y	16-22	NP_BIND	
2bm0_A	20-26	NP_BIND	
2c78_A	19-25	NP_BIND	
2cxx_A	25-31	NP_BIND	
2cxx_A	9-15	-	9,10,11,12,13,14,15
2d0t_A	155-161	-	
2e3b_A	74-80	NP_BIND	
2i1q_A	106-112	NP_BIND	
3tmk_A	13-19	NP_BIND	

Table S5 — Analysis of `EIJU` fragments

Firstly, we compared `EIJU` fragments with Swiss-Prot annotations. Then, for unannotated fragments, we predicted residues involved in a NAD(P)-binding sites using SitePredict software (Bordner et al., 2008). Only residues predicted with a score higher than 0.5 and matching with studied `EIJU`-fragments are indicated.
[1]: pdb codes.

Proteins[1]	Word position	Swiss-Prot Annotation	SitePredict
1a0c_A	388-394	-	-
1aoz_A	535-541	NP_BIND	
1cyd_A	14-20	NP_BIND	
1edo_A	24-30	NP_BIND	
1gee_A	14-20	NP_BIND	
1h5q_A	18-24	-	18,19,20,21,22,23,24
1hdc_A	13-19	NP_BIND	
1iy8_A	20-26	NP_BIND	
1k2w_A	12-18	NP_BIND	
1m93_B	256-262	-	-
1oy5_A	21-27	-	-
1ve9_A	108-114	-	-
1w6g_A	562-568	-	-
1x7g_A	13-19	NP_BIND	
2ae2_A	16-22	-	16,17,18,19,20,21,22

Table S6 — Analysis of UGRU fragments

The structural word UGRU is seen 37 times in the initial data set and is strongly over-represented in the "S-adenosyl-L-methionine-dependent methyltransferase" superfamily (SCOP id=53335). When we restrict the analysis to proteins present in the PDB/UniProt Mapping database (= 1 487 proteins composing the annotation data set), it is seen only 12 times. Only four of these 12 fragments (precision=33%) are annotated as "Binding", resulting in the fact that this word is not defined as functional word. The manual analysis of the functional annotations of the 29 UGRU fragments seen in the initial data set and in the "S-adenosyl-L-methionine-dependent methyltransferase" superfamily through the Swiss-Prot web interface (http://www.uniprot.org/uniprot/) revealed that 12 fragments are actually S-adenosyl-L-methionine (SAH/SAM) binding sites. Furthermore eight of 17 unannotated UGRU fragments are extracted from proteins co-crystallized with SAH/SAM, and the UGRU fragments are actually involved in the binding site. Thus, 69% of the 29 UGRU fragments are indeed involved in SAH/SAM-binding sites.

Firstly, we compared UGRU fragments with Swiss-Prot annotations. Then, for unannotated fragments, we extracted residues involved in the SAH-binding sites using Ligplot (Wallace et al., 1995). Only SAH-binding residues matching with UGRU-fragments are presented.
[1]: pdb codes.

Proteins[1]	Position	Swiss-Prot annotation	Ligand	Residues involved in binding site
1l3i_A	39-45	-	SAH	44,45
1kyz_A	206-212	Binding site		
1nv8_A	127-133	-	SAM-MEQ	129
2gh1_A	45-51	-	-	-
1dus_A	61-67	-	-	-
1nw3_A	161-167	-	SAM	161,163
1ri5_A*	70-76	Binding site		
1rjd_A	103-109	Binding site		
1im8_A	61-67	-	SAI	63,65
2aot_A	58-64	Binding site		
1fp1_D	215-221	Binding site		
2fk8_A	79-85	-	SAM	81,83
2fyt_A	278-284	Binding site		
1nkv_A	42-48	-		
1qyr_A	43-49	Binding site		
1jsx_A	71-77	-	-	-
1ne2_A	54-60	-	-	-
2ex4_A	68-74	Binding site		
1i4w_A	52-58	-	-	-
1or8_A	76-82	Binding site		
1zkd_A	86-92	-	-	-
1y8c_A	44-50	-	-	-
1wzn_A	47-53	-	SAH	49
1dl5_A	81-87	-	SAH	-
1zq9_A	62-68	Binding site		
1vl5_A	48-54	-	-	-
1xxl_A	18-24	Binding site		
1yzh_A	44-50	Binding site		
1qzz_A	188-194	-	SAM	190

Table S7 — Analysis of ZCLH fragments

Firstly, we compared ZCLH fragments with Swiss-Prot annotations. Then, for unannotated fragments, we extracted residues involved in a catalytic site using the CSA database (Porter et al., 1994). Only catalytic site residues matching with ZCLH-fragments are presented.
[1]: pdb codes.

Proteins[1]	position	Annotation	Residues in catalytic site
1auo_A	168-174	Active site	
1c4x_A	235-241	-	235
1fj2_A	169-175	Active site	
1isp_A	133-139	Active site	
1j1i_A	233-239	-	233
1uxo_A	137-143	Active site	
1vlq_A	274-280	-	274
1wm1_A	268-274	Active site	

Table S8 — Computation of a random sensitivity for each functional word

Random sensitivity values for functional word and the associated functional annotation. In order to analyze the significance of sensitivity values obtained for each pair functional word / annotation, we compare these values to ones computed in a a random dataset. The random data set is built by randomly assigning a functional annotation for each word. Using this random data set, we compute the random recall between the each pairs functional word / annotation. We ran this procedure 10,000 times and we compute the average random recall values. [1] and [2]: are computed on the 10,000 simulations.

Word	Swiss-Prot annotation	Sensitivity	Random sensitivity	
			Mean[1]	Standard deviation[2]
DODQ	Calcium-binding sites	95	0.35	1.32
ZDOD	Calcium-binding sites	64	0.27	1.14
YUOD	ATP/GTP-binding sites	29	3.3	1.67
UODO	ATP/GTP-binding sites	40	4.25	1.89
OEIJ	NAD(P)-binding sites	7	1.57	2.02
EIJU	NAD(P)-binding sites	10	2.12	2.33
RUDO	SAM/SAH-binding sites	44	0.7	1.57
